# Evaluation of suitable reference genes for gene expression analysis in the northern root-knot nematode, *Meloidogyne hapla*

**DOI:** 10.1371/journal.pone.0218610

**Published:** 2019-06-19

**Authors:** Xiaojing Wu, Hongyan Yu, Rouwei Yang, Yuanyuan Zhou, Xiaofeng Zhu, Yuanyuan Wang, Xiaoyu Liu, Haiyan Fan, Lijie Chen, Yuxi Duan

**Affiliations:** 1 College of Plant Protection, Shenyang Agricultural University, Shenyang, Liaoning, China; 2 College of Biology Science and Technology, Shenyang Agricultural University, Shenyang, Liaoning, China; 3 College of Science, Shenyang Agricultural University, Shenyang, Liaoning, China; Hainan University, CHINA

## Abstract

The northern root-knot nematode (*Meloidogyne hapla*) is a critical pathogen with a wide host range. Quantitative real-time polymerase chain reaction (qRT-PCR) has been used to elucidate gene expression and function of *M*. *hapla*. Suitable reference genes are required to ensure accurate results of qRT-PCR for normalising gene expression. Eleven candidate reference genes of *M*. *hapla* were selected to evaluate gene expression stability under different conditions. The stability of candidate reference genes was ranked using RefFinder analysis, and the optimal number of reference genes was recommended with geNorm. Notably, the most stable reference genes were *SDHA*, *Mdh*, and *RpS6* for all samples; *SDHA* and *RpS6* were particularly stable during development stage treatments, whereas *Mdh* and *RpS6* were appropriate for temperature and inorganic compound treatments. In contrast, the least stable reference genes were *Actin1* during development stages and all other treatments, *GAPDH* for temperature treatments, and *α-Tub* for inorganic compound treatments. One target gene, *Mh-Hsp90*, was used to verify the selection of reference genes, results showed *Mdh* and *RpS6* could be used as suitable reference genes for *M*. *hapla*, and *Mdh* plus *RpS6* were better. Our finding contributes to further work on gene transcription analysis in *M*. *hapla*.

## Introduction

Quantitative real-time polymerase chain reaction (qRT-PCR) is an important conventional method for measuring gene expression in molecular biology applications and has several advantages, including high sensitivity, wide dynamic range, and low cost [1–4]. However, experimental error can be caused by poor quality and low concentrations of RNA and cDNA [5–10]. In order to reduce error and achieve reliable results, reference genes, called housekeeping genes, are essential for normalising gene expression [11].

Several common reference genes, including arginine kinase (*AK*), Actin 1 (*Actin1*), elongation factor 1 alpha (*EF1-α*), glyceraldehyde-3-phosphate dehydrogenase (*GAPDH*), malate dehydrogenase (*Mdh*), transcriptional activator protein PUR (*Pur*), ribosomal protein S6 (*RpS6*), transcription initiation factor (*TAF*), alpha tubulin (*α-Tub*), polyubiquitin (*Ubp*), and succinate dehydrogenase flavoprotein subunit (*SDHA*), have been identified and used for normalisation of gene expression by qRT-PCR in various organisms [12–17]. An appropriate reference gene shows similar expression levels under different treatments [13]. However, no absolute reference genes in plants or animals or under different experimental treatments have been reported [8, 18–20]. Therefore, identifying the stability of reference genes is a crucial step for qRT-PCR analysis of gene expression. Some specialised analysis software, e.g. geNorm [21], Normfinder [22], and Bestkeeper [23], have been used to evaluate the stability of candidate reference genes under various experimental treatments.

The northern root-knot nematode (*Meloidogyne hapla*) is a sedentary-biotrophic parasite that feeds on plant roots and induces galling. The plant damages caused by nematodes influence the root system and reduce crop yields or quality [24–26], consequently causing severe economic losses in temperature cropping regions [27, 28]. The whole genome sequence of *M*. *hapla* is available, and the size (54 Mb) of the *M*. *hapla* genome is smaller than that (184 Mb) of *M*. *incognita* [29, 30]. Moreover, the proteome of *M*. *hapla* has been annotated [31], and the *MhTTL2* and *Mh265* genes, which are related to parasitism, have been identified [32]. *M*. *hapla* has a moderate cold tolerance [26] and is sensitive to some inorganic compounds [33]. However, the adaptation strategies of *M*. *hapla* remain unknown. Gene expression analysis by qRT-PCR is an important method that monitors expression of certain candidate genes in response to cold temperature and inorganic compound stress in *M*. *hapla*, which might lend insight into mechanisms of nematodes adaptation to certain stress environmental. However, no comprehensive studies of appropriate reference genes in *M*. *hapla* have been performed.

Accordingly, in this study, eleven candidate reference genes in *M*. *hapla* were evaluated to determine their stability for normalisation of gene expression under different experimental treatments (development stages, temperature, and inorganic compounds). The target gene *Mh-Hsp90* was used to identify the selection of reference genes.

## Materials and methods

### Nematode culture and treatments

The northern root-knot nematode *M*. *hapla* was maintained on susceptible tomatoes (L-402) in a greenhouse as described by Forge and MacGuidwin [34]. The eggs were extracted from tomato roots [26]. Second stage juveniles (J2) were collected 48 hr after egg hatching. Females were picked from diseased roots for the experiment.

#### Development stage treatments

*M*. *hapla* eggs, J2 and females were transferred to 1.5-mL Eppendorf tubes and centrifuged. Pellets weighing approximately 20 mg were collected, immediately frozen in liquid nitrogen and stored at −80°C for analysis.

#### Temperature treatments

Approximately 20 mg of centrifuged J2 was collected as described above, transferred to a 30 mm diameter Petri dish containing 4 mL sterile water. The samples were respectively exposed to a low temperature (4°C) for 12 hr, preferred temperature (25°C) for 12 hr, and high temperatures (38°C and 40°C) using a programmable cooling device (TEMI990, Shanghai, China) in a temperature-control chamber. The temperature was initially set at 34°C and then increased in increments of 0.5°C/min to 38°C and 40°C; samples were held for 30 min at the high temperatures. The samples were then cooled to 34°C by decreasing the temperature in increments of 0.5°C/min. After removal of the samples from the temperature chamber, the liquid supernatant was discarded, and the pellets were immediately frozen in liquid nitrogen and stored at −80°C.

#### Inorganic compound treatments

Approximately 20 mg J2 was collected in a 1.5 ml tube and 500 uL inorganic compound (6 mM NH_4_HCO_3_, 0.77 mM FeCl_3_·6H_2_O, 0.16 mM CuCl_2_·2H_2_O, and 0.16 mM CuSO_4_·5H_2_O) was added to each tube [33]. The samples were incubated at 25°C under dark conditions for 24 hr, rinsed for five times with RNase-free water, frozen, and stored at −80°C. Three independent biological replicates were evaluated for each treatment.

### Total RNA isolation and cDNA synthesis

Total RNA was extracted using a MiniBEST Universal RNA Extraction Kit (TaKaRa, Dalian, China) according to the manufacturer’s instructions. The concentration and purity were determined twice for each RNA sample by NanoVue, and samples with an A_260_/A_280_ ratio between 1.9 and 2.2 were used for cDNA synthesis. Five hundred nanogram of RNA was reverse-transcribed into cDNA in a final volume of 10 μL using a PrimeScript RT Master Mix (TaKaRa). The cDNA was serially diluted 10-fold (10×, 10^2^×, 10^3^×, 10^4^×, and 10^5^× dilutions) to assess the amplification efficiency (*E*%) of primers and correlation coefficients (*R*^2^) or 5-fold for qPCR analysis.

### The primer design and qRT-PCR method

The eleven candidate reference genes were *AK*, *Actin1*, *EF1-α*, *GAPDH*, *Mdh*, *Pur*, *RpS6*, *TAF*, *α-Tub*, *Ubp*, and *SDHA*. The EST sequences of candidate genes and the mRNA sequences of the target gene (*Mh-Hsp90*) were obtained from the GenBank database. Twelve pairs of specific primers were designed by Primer Premier 5 according to the design parameters of qPCR primers with 55–65°C melting temperature, 18–23 bp primer length, 30–55% GC content and 90–260 bp product length.

qPCR was performed using SYBR Premix Ex Taq II (TaKaRa) following the manufacturer’s protocol on a Bio-Rad CFX-96 real-time PCR system (Bio-Rad, Hercules, CA, USA). Each 10-μL qPCR mixture included 5 μL SYBR Premix Ex Taq II, 1 μL diluted cDNA, 0.4 μL of each primer (10 μM), and 3.2 μL ddH_2_O. The reaction conditions were as follows: 95°C for 30 s, followed by 40 cycles of 95°C for 5 s and 60°C for 30 s, and a final melt curve from 65°C to 95°C with a 0.5°C increment. Each treatment included three technical and biological replicates.

### Data analysis

The amplification efficiency of primers was calculated using the formula: %E = (10^[-1/slope]^ − 1) × 100% [35], and the correlation coefficient (R^2^ > 0.99) was obtained from the standard curve of a 10-fold dilution template.

Four data analysis methods, the ΔCq method, geNorm [21], Normfinder [22], and Bestkeeper [23], and the website tool RefFinder were used to evaluate the best reference genes. The data were directly analysed with BestKeeper, but converted into relative quantities for geNorm and Normfinder via the formula 2^-ΔCq^, ΔCq = the corresponding Cq value − minimum Cq [36]. The geNorm program calculated the expression stability value (*M*) for each gene, and the pairwise variation (V_n_ / V_n + 1_) to find the optimal number of reference genes. Using stability values, each candidate gene was estimated with NormFinder software. The lowest *M*-value indicated the highest stability. The gene with the highest stability was ranked with BestKeeper based on the standard deviation value and coefficient of variation value. Finally, a web-based analysis tool RefFinder was used to comprehensively estimate the best candidate genes from each program [37–39].

### Analysis of reference gene validation

The expression level of the heat-shock protein 90 gene *(Hsp90*) involved in the regulation of environmental stress, is increased in nematodes under abiotic stresses, such as heat shock stress and inorganic compound stress [40, 41]. Therefore, the expression levels of *Hsp90* in *M*. *hapla* (*Mh-Hsp90*) were evaluated to validate the identified reference genes. The qRT-PCR primer pairs for *Mh-Hsp90* were 5’- TTGCTAAATCTGGCACGAAGG -3’ (forward) and 5’- ATGAAGGAACCACCAGCAGA -3’ (reverse).

## Results

### Primer amplification efficiency and specificity of candidate reference genes

**Fig 1:**
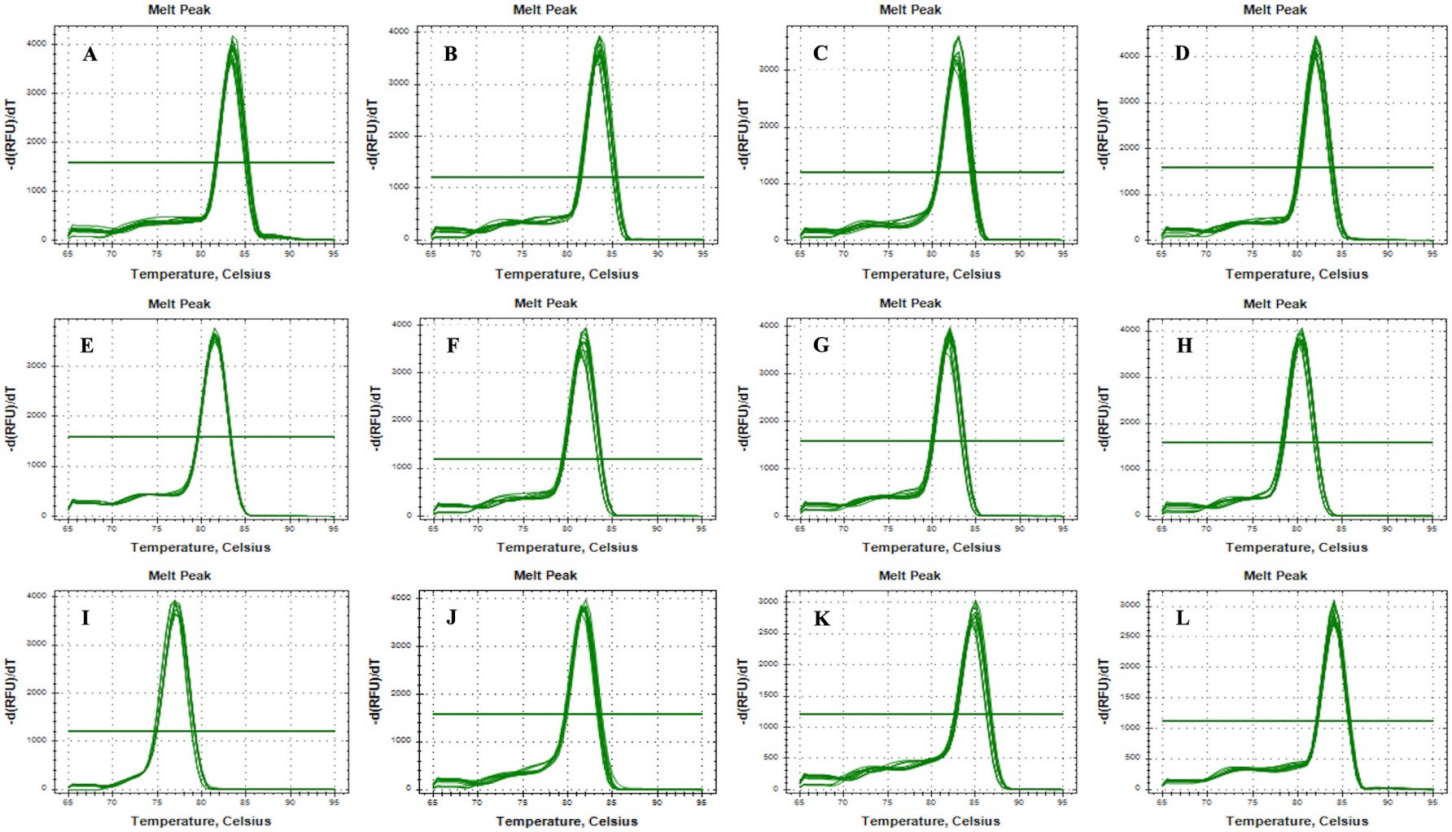
Melting curves for candidate reference genes and target gene. A: *Actin1*, B: *AK*, C: *EF1-α*, D: *GAPDH*, E: *Mdh*, F: *Pur*, G: *RpS6*, H: *TAF*, I: *α-Tub*, J: *Ubp*, K: *SDHA*, L: *Mh-Hsp 90*.

The eleven candidate reference genes with accession numbers, primer pair sequences, annealing temperatures, PCR product lengths, the Tm of products, amplification efficiencies (E%), and correlation coefficients (*R*^2^) are listed in Table 1. The E% values for the eleven candidate reference genes ranged from 93.6% to 106.7%, and the *R*^*2*^ values reached 0.99 (Table 1). A single peak in the melting curve showed specific amplification of all primers (Fig 1).

**Table 1:** Details and primer sequences of candidate reference genes and target gene used in qRT-PCR.

Gene	Accession number	Primer Sequence (5’-3’)	Product length (bp)	E (%)	*R*^2^	the Tm (°C) of products	Annealing temperature (°C) of primers
*Actin 1*	CA996975.1	F:GATGGTGGGAATGGGACAGA	214	100.2	0.999	83.5	63.43
		R:AGCCTTTGGGTTGAGTGGAG					64.21
*AK*	CN577415.1	F:ATCTGCTTCACAGCCTCA	222	104.6	0.999	83.5	59.35
		R:GCTCACTTTTTGCCCTTC					57.72
*EF1-α*	CN576760.1	F:AGCAACGACCAAAACAGC	212	106.6	0.996	83.0	60.00
		R:AGGAAATGGGAAAGGGAT					56.62
*GAPDH*	BQ627356.1	F:ATCGGTCGTCTTGCCTTAC	242	106.3	0.998	82.0	61.1
		R:CCTGCCCAGTCAATCTTTT					59.28
*Mdh*	CA997091.1	F:GAAAGCCAGGGATGACAC	100	96.7	0.999	81.5	58.81
		R:AGAAAAGCATTGGGACAG					55.88
*Pur*	CA997073.1	F:GAGGAGATTGCGAGTAAGTC	144	100.2	0.997	81.5	59.53
		R:AACGAGCATTGTCATAAAAA					55.82
*RpS6*	BQ627371.1	F:CGTGTTCGTCTACTTCTCTCT	153	102.1	0.999	82.0	61.36
		R:TTCAATCTCGTTATCACCTTT					58.40
*TAF*	CN194315.1	F:CGTTTCTGTGACAATGTATGG	143	106.7	0.997	80.5	59.66
		R:GGCTTTCCAAATGGCTCT					58.70
*α-Tub*	CN575322.1	F:CGGCAAACAAGCATGGAG	149	100.9	0.999	77.0	60.55
		R:ATGGATTCGGCTGGTGGA					61.88
*Ubp*	BM900495.1	F:ATTTGGTTCTGCGTCTCCG	135	93.6	0.999	82.0	61.99
		R:GAATGCCCTCTTTGTCCTG					59.75
*SDHA*	CA997448.1	F:GGTCAATCACGACGGGTT	156	101.9	0.999	85.0	60.79
		R:CAGTATGTGAACGAGTAGGAAAC					61.95
*Mh-Hsp 90*	AY528417	F:TTGCTAAATCTGGCACGAAGG	255	102.1	0.997	85	62.49
		R:ATGAAGGAACCACCAGCAGAA					61.96

### Cq value analysis of candidate reference genes

**Fig 2:**
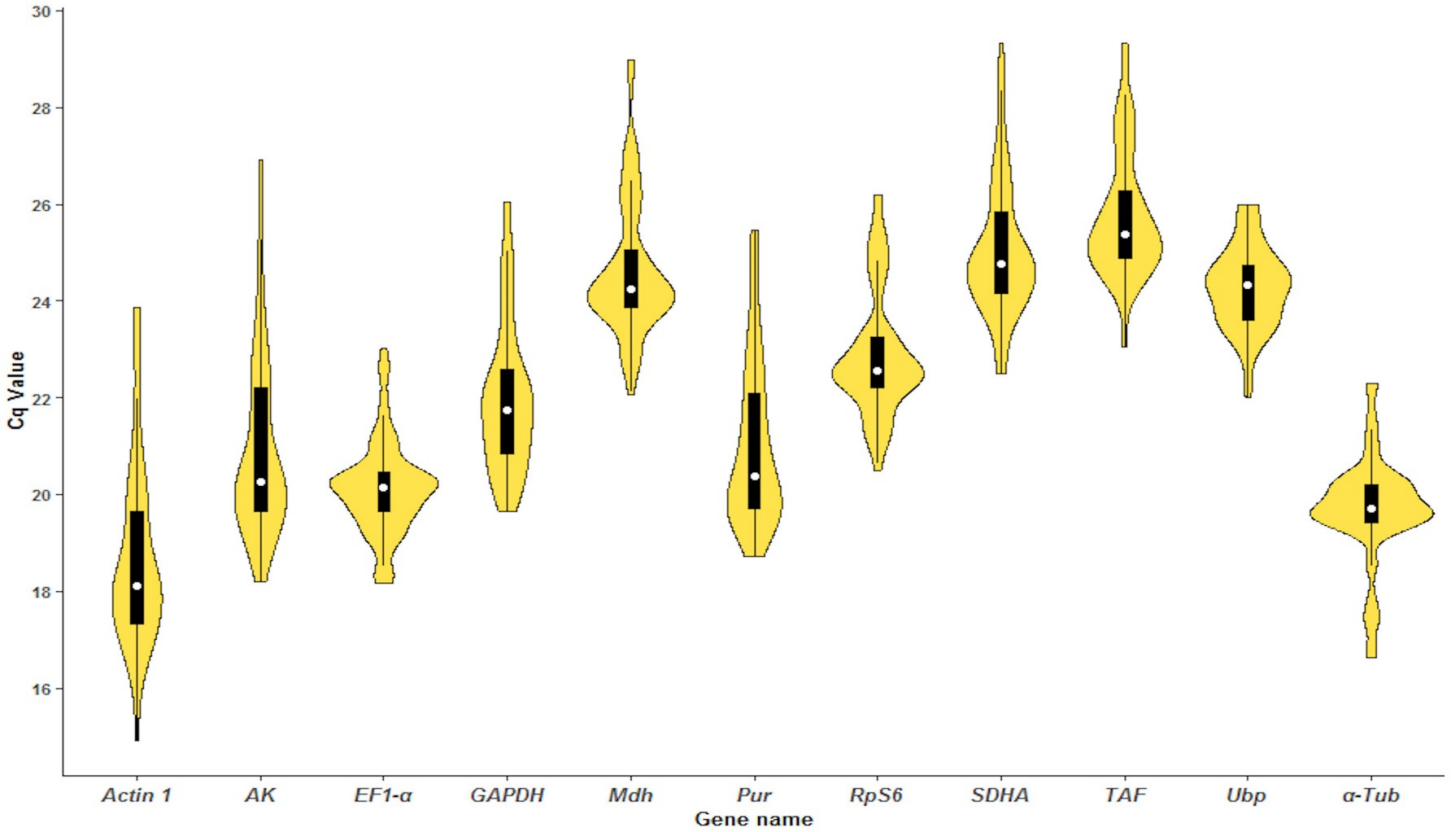
The distributions of all raw Cq values for eleven candidate reference genes in all samples of *Meloidogyne hapla*. The open circle inside the boxes represents the median. The black bold lines present the 25^th^ and 75^th^ percentiles. The thin lines indicate the 5^th^ and 95^th^ percentiles.

Cq values indicate the expression levels of reference genes. The distributions of all Cq values for all samples are shown in Fig 2. The Cq values ranged from 14.91 to 29.34 for the eleven candidate reference genes, and the mean values for *Actin1* and *TAF* were 18.59 and 25.7, respectively. Low Cq values indicate high expression levels. Among the eleven reference genes, *Actin1* showed high expression, whereas *TAF* showed low expression.

### Expression stability of candidate reference genes under different treatments

Five methods (ΔCq method, geNorm, NormFinder, BestKeeper, and RefFinder) were used to evaluated the stability of eleven candidate reference genes. Each reference gene was subjected to ten treatments, and the stability of them analysed individually (Table 2). In addition, these ten treatments were divide into four groups for more comprehensive analysis: “Development stage (egg, J2 and female), Temperature treatments (4, 25, 38 and 40°C), Inorganic compound treatments (CuSO_4_·5H_2_O, FeCl_3_·6H_2_O, CuCl_2_·2H_2_O, and NH_4_HCO_3_), and All treatments (composed of all the treatments sets). The ranks of the eleven genes for groups were calculated and shown in Table 2.

**Table 2:** Stability of candidate reference genes under different treatments.

Treatments	Rank	ΔCq method		geNorm		NormFinder		BestKeeper		RefFinder	
		Gene name	Stability value	Gene name	Stability value	Gene name	Stability value	Gene name	Stability value	Gene name	Geomean of ranking values
4°C	1	*Pur*	0.47	*Mdh | RpS6*	0.050	*Pur*	0.121	*Pur*	0.189	*Pur*	1.50
	2	*Mdh*	0.49			*Mdh*	0.178	*Mdh*	0.198	*Mdh*	1.86
	3	*RpS6*	0.51	*SDHA*	0.204	*GAPDH*	0.209	*RpS6*	0.202	*RpS6*	2.21
	4	*GAPDH*	0.54	*Actin 1*	0.250	*RpS6*	0.226	*α-Tub*	0.246	*GAPDH*	4.56
	5	*α-Tub*	0.58	*Pur*	0.306	*α-Tub*	0.351	*TAF*	0.300	*α-Tub*	5.14
	6	*SDHA*	0.60	*GAPDH*	0.373	*SDHA*	0.449	*GAPDH*	0.344	*SDHA*	5.24
	7	*AK*	0.67	*α-Tub*	0.431	*AK*	0.510	*SDHA*	0.404	*Actin 1*	6.93
	8	*Actin 1*	0.68	*AK*	0.484	*TAF*	0.590	*Actin 1*	0.464	*TAF*	7.54
	9	*TAF*	0.74	*TAF*	0.523	*Actin 1*	0.599	*AK*	0.480	*AK*	7.71
	10	*Ubp*	0.81	*Ubp*	0.564	*Ubp*	0.770	*Ubp*	0.604	*Ubp*	10.00
	11	*EF1-α*	1.01	*EF1-α*	0.645	*EF1-α*	0.968	*EF1-α*	0.800	*EF1-α*	11.00
25°C	1	*α-Tub*	0.33	*GAPDH | TAF*	0.059	*α-Tub | RpS6*	0.049	*Pur*	0.078	*α-Tub*	2.21
	2	*AK*	0.33					*AK*	0.080	*AK*	2.78
	3	*RpS6*	0.33	*EF1-α*	0.095	*AK*	0.085	*GAPDH*	0.156	*Pur*	2.83
	4	*Pur*	0.36	*Pur*	0.149	*Pur*	0.202	*α-Tub*	0.160	*GAPDH*	3.35
	5	*Mdh*	0.38	*AK*	0.182	*Mdh*	0.230	*TAF*	0.184	*RpS6*	4.14
	6	*GAPDH*	0.44	*α-Tub*	0.238	*Actin 1*	0.338	*EF1-α*	0.200	*TAF*	4.23
	7	*Actin 1*	0.45	*RpS6*	0.273	*GAPDH*	0.399	*RpS6*	0.204	*Mdh*	6.32
	8	*TAF*	0.47	*Mdh*	0.314	*TAF*	0.443	*Mdh*	0.309	*EF1-α*	6.34
	9	*EF1-α*	0.50	*Actin 1*	0.351	*SDHA*	0.458	*Actin 1*	0.342	*Actin 1*	7.64
	10	*SDHA*	0.51	*SDHA*	0.390	*EF1-α*	0.476	*SDHA*	0.447	*SDHA*	9.74
	11	*Ubp*	0.60	*Ubp*	0.428	*Ubp*	0.581	*Ubp*	0.540	*Ubp*	11.00
38°C	1	*Mdh*	0.61	*AK | Actin 1*	0.046	*Pur*	0.138	*Ubp*	0.098	*Mdh*	2.78
	2	*SDHA*	0.61			*Mdh*	0.169	*EF1-α*	0.402	*Pur*	3.60
	3	*RpS6*	0.61	*TAF*	0.078	*RpS6*	0.323	*Pur*	0.513	*SDHA*	3.87
	4	*TAF*	0.62	*SDHA*	0.106	*SDHA*	0.398	*α-Tub*	0.636	*RpS6*	4.05
	5	*Actin 1*	0.66	*RpS6*	0.135	*TAF*	0.435	*Mdh*	0.684	*Actin 1*	4.05
	6	*AK*	0.68	*Mdh*	0.160	*Actin 1*	0.538	*RpS6*	0.749	*AK*	4.53
	7	*Pur*	0.68	*GAPDH*	0.222	*AK*	0.545	*SDHA*	0.804	*TAF*	4.68
	8	*GAPDH*	0.88	*Pur*	0.285	*Ubp*	0.811	*TAF*	0.838	*Ubp*	5.05
	9	*Ubp*	1.08	*Ubp*	0.487	*GAPDH*	0.840	*Actin 1*	0.902	*EF1-α*	6.69
	10	*EF1-α*	1.36	*EF1-α*	0.682	*EF1-α*	1.256	*AK*	0.909	*α-Tub*	8.54
	11	*α-Tub*	1.66	*α-Tub*	0.860	*α-Tub*	1.641	*GAPDH*	1.076	*GAPDH*	8.63
40°C	1	*Mdh*	0.17	*AK | RpS6*	0.038	*Mdh*	0.031	*Actin 1*	0.104	*Mdh*	2.06
	2	*RpS6*	0.18			*RpS6*	0.067	*α-Tub*	0.129	*RpS6*	2.11
	3	*EF1-α*	0.18	*Mdh*	0.097	*EF1-α*	0.087	*AK*	0.149	*AK*	2.63
	4	*AK*	0.19	*EF1-α*	0.112	*AK*	0.098	*EF1-α*	0.162	*EF1-α*	3.46
	5	*SDHA*	0.20	*SDHA*	0.137	*SDHA*	0.128	*RpS6*	0.178	*Actin 1*	5.33
	6	*Pur*	0.21	*Pur*	0.151	*Pur*	0.160	*Mdh*	0.198	*SDHA*	5.62
	7	*GAPDH*	0.23	*GAPDH*	0.156	*GAPDH*	0.180	*Ubp*	0.202	*α-Tub*	6.51
	8	*Ubp*	0.26	*Ubp*	0.177	*Ubp*	0.218	*SDHA*	0.291	*Pur*	6.82
	9	*Actin 1*	0.27	*α-Tub*	0.194	*Actin 1*	0.226	*TAF*	0.304	*Ubp*	7.74
	10	*α-Tub*	0.27	*Actin 1*	0.207	*α-Tub*	0.242	*Pur*	0.307	*GAPDH*	7.84
	11	*TAF*	0.28	*TAF*	0.221	*TAF*	0.252	*GAPDH*	0.329	*TAF*	10.46
CuSO_4_**·**5H_2_O	1	*Actin 1*	0.57	*Mdh | RpS6*	0.035	*Pur*	0.205	*EF1-α*	0.222	*Pur*	2.30
	2	*Pur*	0.60			*Actin 1*	0.207	*GAPDH*	0.236	*Actin 1*	2.91
	3	*RpS6*	0.60	*SDHA*	0.060	*RpS6*	0.478	*Pur*	0.238	*RpS6*	2.91
	4	*Mdh*	0.61	*Ubp*	0.098	*AK*	0.489	*α-Tub*	0.242	*Mdh*	3.66
	5	*SDHA*	0.65	*AK*	0.189	*Mdh*	0.508	*TAF*	0.258	*EF1-α*	5.05
	6	*AK*	0.66	*Actin 1*	0.235	*SDHA*	0.576	*Actin 1*	0.518	*AK*	5.38
	7	*Ubp*	0.73	*Pur*	0.344	*GAPDH*	0.597	*AK*	0.664	*SDHA*	5.48
	8	*GAPDH*	0.76	*GAPDH*	0.493	*EF1-α*	0.659	*RpS6*	0.691	*GAPDH*	6.05
	9	*EF1-α*	0.78	*EF1-α*	0.581	*Ubp*	0.679	*Mdh*	0.713	*Ubp*	7.26
	10	*TAF*	0.89	*TAF*	0.661	*TAF*	0.837	*SDHA*	0.756	*TAF*	8.41
	11	*α-Tub*	0.91	*α-Tub*	0.706	*α-Tub*	0.841	*Ubp*	0.822	*α-Tub*	8.54
FeCl_3_**·**6H_2_O	1	*RpS6*	0.25	*Pur / RpS6*	0.058	*RpS6*	0.029	*Ubp*	0.049	*RpS6*	1.57
	2	*TAF*	0.26			*Actin 1*	0.044	*Actin 1*	0.116	*Actin 1*	2.99
	3	*Mdh*	0.26	*Mdh*	0.071	*TAF*	0.046	*EF1-α*	0.178	*TAF*	3.13
	4	*Actin 1*	0.27	*TAF*	0.098	*Mdh*	0.064	*TAF*	0.182	*Mdh*	3.66
	5	*Pur*	0.27	*Actin 1*	0.116	*Ubp*	0.109	*Mdh*	0.209	*Pur*	3.81
	6	*Ubp*	0.31	*SDHA*	0.140	*Pur*	0.117	*RpS6*	0.211	*Ubp*	3.81
	7	*SDHA*	0.32	*Ubp*	0.162	*SDHA*	0.196	*Pur*	0.256	*EF1-α*	6.82
	8	*AK*	0.41	*AK*	0.203	*EF1-α*	0.348	*SDHA*	0.264	*SDHA*	6.96
	9	*EF1-α*	0.44	*GAPDH*	0.242	*AK*	0.348	*AK*	0.338	*AK*	8.49
	10	*GAPDH*	0.49	*EF1-α*	0.283	*GAPDH*	0.464	*α-Tub*	0.364	*GAPDH*	9.97
	11	*α-Tub*	0.74	*α-Tub*	0.366	*α-Tub*	0.730	*GAPDH*	0.469	*α-Tub*	10.74
CuCl_2_·2H_2_O	1	*Mdh*	0.60	*GAPDH | TAF*	0.081	*RpS6*	0.070	*EF1-α*	0.202	*TAF*	2.78
	2	*TAF*	0.61			*SDHA*	0.166	*Ubp*	0.598	*RpS6*	2.91
	3	*RpS6*	0.61	*Actin 1*	0.106	*Ubp*	0.209	*SDHA*	0.702	*Mdh*	3.25
	4	*GAPDH*	0.64	*Mdh*	0.135	*Mdh*	0.321	*RpS6*	0.871	*GAPDH*	3.72
	5	*Pur*	0.65	*Pur*	0.149	*TAF*	0.343	*α-Tub*	0.902	*SDHA*	4.28
	6	*Actin 1*	0.68	*RpS6*	0.179	*GAPDH*	0.447	*TAF*	0.971	*Ubp*	4.56
	7	*SDHA*	0.72	*AK*	0.235	*Pur*	0.470	*Mdh*	0.976	*EF1-α*	5.62
	8	*Ubp*	0.82	*SDHA*	0.295	*Actin 1*	0.546	*GAPDH*	1.020	*Actin 1*	6.16
	9	*AK*	0.84	*Ubp*	0.376	*AK*	0.747	*Pur*	1.060	*Pur*	6.30
	10	*EF1-α*	1.39	*EF1-α*	0.588	*EF1-α*	1.159	*Actin 1*	1.084	*AK*	8.89
	11	*α-Tub*	2.27	*α-Tub*	0.894	*α-Tub*	2.261	*AK*	1.224	*α-Tub*	9.03
NH_4_HCO_3_	1	*TAF*	0.20	*TAF | SDHA*	0.044	*TAF*	0.022	*AK*	0.129	*TAF*	1.32
	2	*Mdh*	0.21			*Mdh*	0.031	*Actin 1*	0.193	*Actin 1*	2.91
	3	*SDHA*	0.21	*Actin 1*	0.046	*Actin 1*	0.036	*TAF*	0.211	*SDHA*	2.94
	4	*Actin 1*	0.21	*Pur*	0.056	*RpS6*	0.066	*Mdh*	0.218	*Mdh*	2.99
	5	*RpS6*	0.22	*Mdh*	0.073	*SDHA*	0.069	*SDHA*	0.231	*AK*	4.76
	6	*Pur*	0.23	*RpS6*	0.086	*Pur*	0.114	*EF1-α*	0.258	*RpS6*	5.18
	7	*Ubp*	0.30	*Ubp*	0.126	*Ubp*	0.199	*Pur*	0.260	*Pur*	5.63
	8	*AK*	0.36	*AK*	0.168	*AK*	0.292	*RpS6*	0.262	*Ubp*	7.45
	9	*EF1-α*	0.37	*GAPDH*	0.207	*EF1-α*	0.301	*Ubp*	0.340	*EF1-α*	8.97
	10	*GAPDH*	0.42	*EF1-α*	0.243	*GAPDH*	0.402	*α-Tub*	0.351	*GAPDH*	9.97
	11	*α-Tub*	0.55	*α-Tub*	0.298	*α-Tub*	0.531	*GAPDH*	0.422	*α-Tub*	10.74
Egg	1	*Actin 1*	0.73	*Mdh | TAF*	0.107	*Pur*	0.077	*α-Tub*	0.358	*Mdh*	2.99
	2	*Mdh*	0.75			*SDHA*	0.300	*Ubp*	0.527	*Pur*	3.03
	3	*Pur*	0.77	*RpS6*	0.137	*GAPDH*	0.342	*EF1-α*	0.736	*Actin 1*	3.25
	4	*SDHA*	0.77	*Actin 1*	0.162	*Actin 1*	0.433	*Pur*	1.351	*SDHA*	3.94
	5	*GAPDH*	0.79	*SDHA*	0.222	*Mdh*	0.551	*GAPDH*	1.436	*GAPDH*	4.61
	6	*RpS6*	0.80	*GAPDH*	0.277	*RpS6*	0.619	*SDHA*	1.476	*TAF*	4.70
	7	*TAF*	0.81	*Pur*	0.302	*TAF*	0.674	*Actin 1*	1.571	*RpS6*	5.58
	8	*AK*	0.94	*AK*	0.330	*AK*	0.865	*Mdh*	1.640	*α-Tub*	5.62
	9	*Ubp*	1.29	*Ubp*	0.574	*Ubp*	0.970	*RpS6*	1.664	*Ubp*	6.18
	10	*α-Tub*	1.52	*α-Tub*	0.775	*α-Tub*	1.325	*TAF*	1.718	*EF1-α*	7.95
	11	*EF1-α*	2.19	*EF1-α*	1.033	*EF1-α*	2.159	*AK*	1.840	*AK*	8.66
Female	1	*Actin 1*	0.94	*GAPDH | SDHA*	0.087	*AK*	0.227	*Pur*	0.671	*Actin 1*	2.91
	2	*RpS6*	0.95			*Actin 1*	0.302	*Ubp*	0.987	*AK*	3.03
	3	*AK*	0.97	*RpS6*	0.139	*Ubp*	0.474	*EF1-α*	1.044	*RpS6*	3.81
	4	*TAF*	1.01	*Mdh*	0.156	*Pur*	0.538	*AK*	1.047	*Pur*	4.00
	5	*GAPDH*	1.03	*TAF*	0.205	*RpS6*	0.716	*α-Tub*	1.138	*GAPDH*	4.43
	6	*SDHA*	1.03	*Actin 1*	0.280	*TAF*	0.777	*Actin 1*	1.313	*SDHA*	4.68
	7	*Mdh*	1.05	*AK*	0.355	*GAPDH*	0.846	*RpS6*	1.382	*Ubp*	4.70
	8	*Pur*	1.23	*Pur*	0.530	*SDHA*	0.888	*TAF*	1.382	*TAF*	5.57
	9	*Ubp*	1.26	*Ubp*	0.674	*Mdh*	0.931	*Mdh*	1.489	*Mdh*	6.90
	10	*EF1-α*	2.30	*EF1-α*	1.048	*EF1-α*	2.253	*SDHA*	1.489	*EF1-α*	7.40
	11	*α-Tub*	2.33	*α-Tub*	1.281	*α-Tub*	2.285	*GAPDH*	1.516	*α-Tub*	9.03
Development stage	1	*SDHA*	1.24	*Mdh | SDHA*	0.37	*RpS6*	0.503	*Ubp*	0.901	*SDHA*	1.93
	2	*RpS6*	1.28			*SDHA*	0.547	*EF1-α*	1.027	*RpS6*	2.51
	3	*Mdh*	1.3	*GAPDH*	0.474	*Mdh*	0.753	*α-Tub*	1.108	*Mdh*	2.71
	4	*GAPDH*	1.35	*RpS6*	0.55	*Ubp*	0.767	*TAF*	1.324	*Ubp*	3.98
	5	*Pur*	1.51	*AK*	0.824	*GAPDH*	0.836	*RpS6*	1.462	*GAPDH*	4.68
	6	*TAF*	1.52	*Actin 1*	0.929	*Pur*	0.961	*Mdh*	1.637	*TAF*	6.05
	7	*Ubp*	1.52	*Pur*	0.973	*TAF*	1.009	*SDHA*	1.683	*Pur*	6.59
	8	*Actin 1*	1.65	*TAF*	1.043	*Actin 1*	1.327	*GAPDH*	1.871	*EF1-α*	6.69
	9	*AK*	1.65	*Ubp*	1.14	*AK*	1.372	*Pur*	1.994	*α-Tub*	7.95
	10	*EF1-α*	2.34	*EF1-α*	1.409	*EF1-α*	2.087	*AK*	2.208	*AK*	7.98
	11	*α-Tub*	2.69	*α-Tub*	1.641	*α-Tub*	2.545	*Actin 1*	2.266	*Actin 1*	8.06
Temperature	1	*Mdh*	0.63	*Mdh | RpS6*	0.131	*Pur*	0.307	*α-Tub*	0.326	*Mdh*	1.97
	2	*RpS6*	0.63			*AK*	0.314	*Ubp*	0.537	*RpS6*	2.74
	3	*AK*	0.67	*SDHA*	0.196	*Mdh*	0.324	*EF1-α*	0.577	*AK*	3.31
	4	*Pur*	0.69	*Actin 1*	0.241	*RpS6*	0.341	*AK*	0.836	*Pur*	3.72
	5	*SDHA*	0.7	*AK*	0.367	*SDHA*	0.453	*Mdh*	0.848	*SDHA*	4.61
	6	*Actin 1*	0.77	*Pur*	0.439	*TAF*	0.549	*SDHA*	0.874	*α-Tub*	6.04
	7	*TAF*	0.79	*TAF*	0.491	*Actin 1*	0.579	*RpS6*	0.88	*Ubp*	6.18
	8	*GAPDH*	0.87	*GAPDH*	0.531	*GAPDH*	0.705	*Pur*	0.884	*Actin 1*	6.24
	9	*Ubp*	0.99	*Ubp*	0.64	*Ubp*	0.801	*Actin 1*	0.906	*TAF*	7.36
	10	*EF1-α*	1.12	*EF1-α*	0.748	*EF1-α*	0.98	*TAF*	0.96	*EF1-α*	7.4
	11	*α-Tub*	1.14	*α-Tub*	0.818	*α-Tub*	1.013	*GAPDH*	1.093	*GAPDH*	8.66
Inorganic compound	1	*RpS6*	0.53	*Mdh | RpS6*	0.083	*RpS6*	0.269	*EF1-α*	0.458	*RpS6*	1.63
	2	*Mdh*	0.54			*Pur*	0.281	*α-Tub*	0.485	*Mdh*	2.71
	3	*Pur*	0.56	*SDHA*	0.166	*Mdh*	0.311	*Ubp*	0.561	*SDHA*	4.12
	4	*SDHA*	0.58	*AK*	0.252	*SDHA*	0.335	*GAPDH*	0.622	*Pur*	4.36
	5	*Actin 1*	0.62	*Actin 1*	0.293	*TAF*	0.407	*TAF*	0.657	*EF1-α*	5.62
	6	*AK*	0.63	*Pur*	0.326	*Actin 1*	0.413	*SDHA*	0.658	*Actin 1*	5.89
	7	*TAF*	0.66	*TAF*	0.393	*GAPDH*	0.448	*RpS6*	0.694	*TAF*	5.92
	8	*GAPDH*	0.68	*GAPDH*	0.434	*AK*	0.469	*Actin 1*	0.723	*GAPDH*	6.51
	9	*Ubp*	0.77	*Ubp*	0.491	*Ubp*	0.575	*Mdh*	0.723	*AK*	6.78
	10	*EF1-α*	0.9	*EF1-α*	0.582	*EF1-α*	0.745	*Pur*	0.732	*Ubp*	6.84
	11	*α-Tub*	1.23	*α-Tub*	0.701	*α-Tub*	1.18	*AK*	0.775	*α-Tub*	7.18
All	1	*SDHA*	0.98	*Mdh | SDHA*	0.314	*SDHA*	0.419	*EF1-α*	0.659	*SDHA*	1.63
	2	*Mdh*	0.98			*RpS6*	0.448	*Ubp*	0.669	*Mdh*	2.45
	3	*RpS6*	1.01	*RpS6*	0.387	*Mdh*	0.458	*α-Tub*	0.698	*RpS6*	3.08
	4	*GAPDH*	1.11	*GAPDH*	0.522	*Ubp*	0.656	*TAF*	0.976	*Ubp*	4.36
	5	*Ubp*	1.18	*Pur*	0.736	*GAPDH*	0.673	*RpS6*	0.984	*GAPDH*	5.03
	6	*TAF*	1.18	*AK*	0.813	*TAF*	0.759	*Mdh*	1.058	*EF1-α*	5.62
	7	*Pur*	1.22	*Actin 1*	0.84	*Pur*	0.875	*SDHA*	1.065	*TAF*	5.83
	8	*AK*	1.29	*TAF*	0.893	*Actin 1*	1.057	*GAPDH*	1.151	*Pur*	6.85
	9	*Actin 1*	1.3	*Ubp*	0.944	*AK*	1.063	*Pur*	1.381	*α-Tub*	7.95
	10	*EF1-α*	1.64	*EF1-α*	1.103	*EF1-α*	1.412	*Actin 1*	1.475	*AK*	8.3
	11	*α-Tub*	1.96	*α-Tub*	1.26	*α-Tub*	1.845	*AK*	1.505	*Actin 1*	8.43

#### Development stage treatments

**Fig 3:**
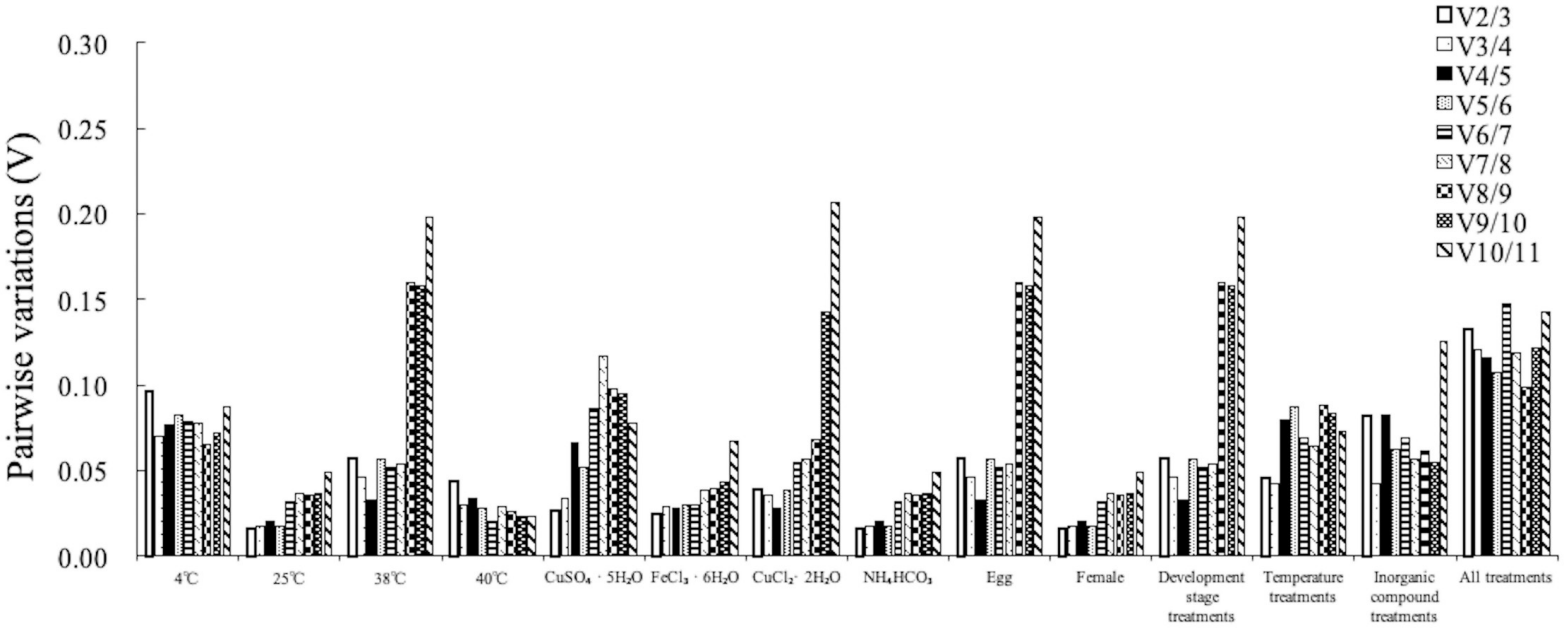
The optimal number of reference genes in *M*. *hapla*. Pairwise variations (V_n/n+1_) were calculated using the geNorm program to determine the optimal number of reference genes for normalization. A value of V_n/n+1_ below 0.15 denoted that additional reference genes were not necessary for improving normalization.

*Mdh* was the stable gene used by ΔCq method, geNorm and RefFingder in egg treatments; The stable gene was *Actin 1* in female treatments and *α-Tub* in J2 treatments through ΔCq method, NormFinder and RefFingder. For the groups, *SDHA*, *RpS6* and *Mdh* was identified as the stable gene by the ΔCq method and Normfinder; *Mdh* and *SDHA* were identified as the most stable genes by geNorm; and *Ubp*, *EF1-α*, and *α-Tub* were identified as the most stable genes by BestKeeper. Therefore, combining all four rankings by RefFinder, *SDHA*, *RpS6*, and *Mdh* were considered the most stable genes, and *α-Tub*, *AK*, and *Actin1* were considered the least stable genes (Table 2). The optimal number of reference genes defined by geNorm shown in Fig 3. The V_2/3_ values was less than 0.15 among egg, female, J2and development stage treatments. Therefore, the best reference gene combination was *Mdh* and *TAF*, *GAPDH* and *SDHA*, *GAPDH* and *TAF*, *Mdh* and *SDHA*, respectively.

#### Temperature treatments

*Pur* and *Mdh* were the most stable gene in 4°C treatment by ΔCq method, NormFinder, BestKeeper and RefFingder. *Mdh* was the most stable gene in 38 and 40°C treatments by ΔCq method, NormFinder, and RefFingder; For the temperature treatments, *EF1-α* and *α-Tub* were identified as the least stable genes by the other three analysis programs except BestKeeper. *Mdh* and *RpS6* were the most stable genes identified by the ΔCq method and geNorm analysis, and *Pur*, *AK*, and *Mdh* showed stable expression in Normfinder analysis. In contrast, *α-Tub*, *Ubp*, and *EF1-α* were identified as stable genes using BestKeeper analysis. Taken together, these results suggested that *Mdh* and *RpS6*, were the most stable genes, whereas *EF1-α*, and *GAPDH* were the least stable genes by RefFinder (Table 2). The V_2/3_ with values lower than 0.15 calculated by geNorm shown in Fig 3 demonstrated that *Mdh* and *RpS6*, *AK* and *Actin 1*, *AK* and *RpS6*, *Mdh* and *RpS6* were sufficient for normalisation among 4°C, 38°C, 40°C and temperature treatments.

#### Inorganic compound treatments

*Pur* and Actin *1* was identified as the most stable gene in CuSO_4_·5H_2_O treatments by ΔCq method, NormFinder, and RefFingder; The most stable gene in FeCl_3_·6H_2_O treatments was *RpS6* used ΔCq method, geNorm, NormFind and RefFingder; *TAF* was the most stable gene in CuCl_2_·2H_2_O and NH_4_HCO_3_ treatments through ΔCq method, geNorm and RefFingderh. The four analysis programs, except for BestKeeper, found that *RpS6* was the most stable gene, and *EF1-α* and *α-Tub* were the least stable genes. Combining ranking demonstrated that *RpS6* and *Mdh* were the most stable genes, whereas *Ubp*, and *α-Tub* were the least stable genes (Table 2). For geNorm, the V_2/3_ values was below 0.15 shown that *Mdh* and *RpS6*, *Pur* and *RpS6*, *GAPDH* and *TAF*, *TAF* and *SDHA*, *Mdh* and *RpS6* were sufficient for normalisation within CuSO_4_·5H_2_O, FeCl_3_·6H_2_O, CuCl_2_·2H_2_O, NH_4_HCO_3_ and inorganic compound treatments (Fig 3).

#### All treatments

Based on the comprehensive ranking, *SDHA*, *Mdh*, and *RpS6* were the most stable genes, as evaluated under all treatments, with geometric mean ranking values of 1.63, 2.45, and 3.08, respectively. In contrast, *α-Tub*, *AK*, and *Actin1* were the least stable genes, with values of 7.95, 8.30, and 8.43, respectively (Table 2). V_2/3_ values of less than 0.15 indicated that two reference genes, *SDHA* and *Mdh*, were sufficient for normalisation in all treatments (Fig 3).

### Validation of stable reference genes

**Fig 4:**
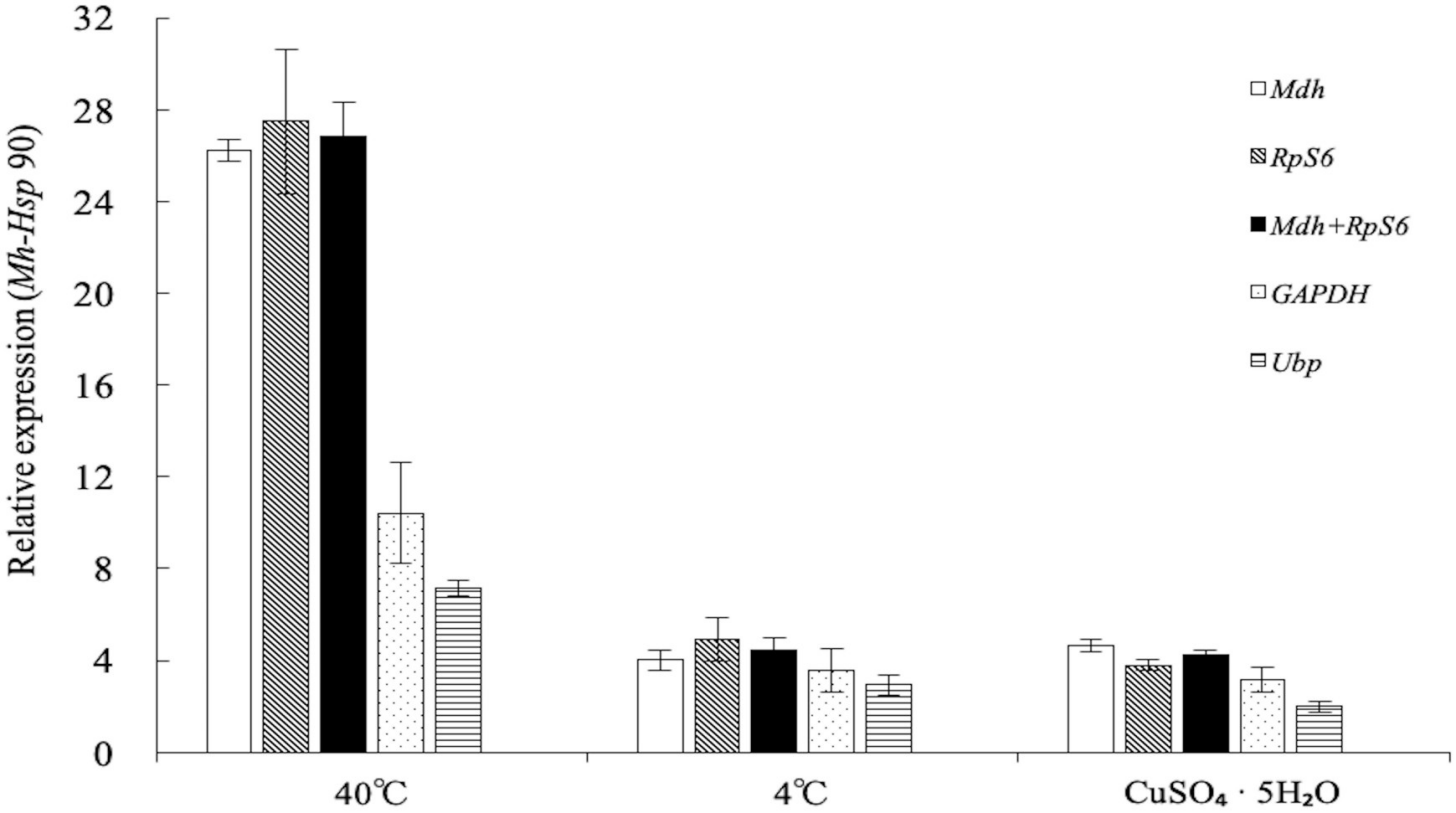
Relative expression of *Mh-Hsp 90* for *M*. *hapla* under different treatments normalized using various reference genes. *Mdh*, *RpS6*, *GAPDH*, *Ubp* and the group of *Mdh* + *RpS6* were used for normalization of *Mh-Hsp 90* gene expression. The bars are means ± standard errors of three technical and biological replicates.

The stable genes *Mdh* and *RpS6*, the unstable gene *GAPDH* and *Ubp*, and the combined group of *Mdh* + *RpS6* were selected to normalise the expression of *Mh-Hsp90* under temperature (40°C and 4°C) and inorganic compound (CuSO_4_·5H_2_O) treatments. Generally, the use of multiple reference genes presents more accurate normalization of the gene expression. Results showed in Fig 4, the relative expression levels of *Mh-Hsp90* were similar when normalised using *Mdh*, *RpS6*, and *Mdh* + *RpS6*, but different when normalised using *GADPH* an*d Ubp*.

## Discussion

qRT-PCR is a powerful technique with high sensitivity and specificity and enables gene expression analysis within a large dynamic range [1, 42, 43]. The amplification efficiency (E%) of reference genes should be similar to that of the target gene, which are essential for improving the accuracy of gene expression. Additionally, an optimal reference gene should show moderate and stable expression levels in all test samples. In our study, the amplification efficiencies (E%) of stable candidate reference genes were similar to that of *Mh-Hsp90* on the same treatments. Moreover, except for *Actin1*, *α-Tub*, and *TAF*, all other genes showed moderate Cq values, and all candidate reference gene were specifically amplified. The values of candidate reference genes were slight difference between programs (geNorm, NormFinder, Bestkeeper) under different treatments, that caused by the difference of algorithms employed [22]. Therefore, it is better to evaluate reference gene using multiple methods, and then determined the suitable reference genes with the geometric mean of comprehensive ranking for all programs which generated by RefFinder.

The stable reference genes have been identified in *Caenorhabditis elegans* that were *tba-1*, *Y45F10D*.*4* and *pmp-3* for studying nanoparticle-induced genetic response [44], *cdc-42*, *pmp-3* and *Y45F10D*.*4* for normalizing 5 *sod* expression levels [45]. In this paper, the *Mdh* and *RpS6* were reliable gene in *M*. *hapla*, consistent with the *RPS15* in *Helicoverpa armigera* [37], *RPS4* in turbot [46], *RpS6* in *Macrobrachium olfersii* [47] for different developmental stages, and similar with the *RPS20* for *Sesamia inferens* [7], *RPS15* and *RPS27* for *H*. *armigera* [37] under different temperature treatments. *EF1-α*, and *GAPDH* were not good reference genes in *M*. *hapla* for temperature stress, while *EF1-α* was useful reference gene for parsley [39]. According to these findings, there were no absolute reference genes for different species and treatments. While, the qRT-PCR relied on accurate normalization of stable reference genes. Therefore, the stability of reference genes should be validated for different experimental condition before use.

Inorganic compounds affect the survival of J2 and the hatch rate of egg-masses for *M*. *hapla* [33]. The responses of *M*. *hapla* to inorganic compound stress are still unknown. Analysis of gene expression will enable researchers to study the effects of inorganic compound stresses on *M*. *hapla*. In this study, we identified suitable reference genes (*RpS6* and *Mdh*) for normalising gene expression. These findings are expected to facilitate further analyses of the mechanisms of inorganic compound stress in *M*. *hapla*.

In conclusions, this work validated that *RpS6*, *Mdh*, *SDHA* and *Pur* could be used as suitable reference genes for normalising qRT-PCR data in *M*. *hapla* under different treatments, and the combination of *RpS6* + *Mdh* were better. This study provides a basis for future studies of gene function in *M*. *hapla*.
